# COVID-19 pandemic sheds a new research spotlight on antiviral potential of essential oils – A bibliometric study

**DOI:** 10.1016/j.heliyon.2023.e17703

**Published:** 2023-06-29

**Authors:** Binawati Ginting, Williams Chiari, Teuku Fais Duta, Syihaabul Hudaa, Agnia Purnama, Harapan Harapan, Diva Rayyan Rizki, Kana Puspita, Rinaldi Idroes, Meriatna Meriatna, Muhammad Iqhrammullah

**Affiliations:** a Department of Chemistry, Faculty of Mathematics and Natural Sciences, Universitas Syiah Kuala, Banda Aceh, 23111, Indonesia; b Department of Mathematics, Faculty of Mathematics and Natural Sciences, Universitas Syiah Kuala, Banda Aceh, 23111, Indonesia; c Innovative Sustainability Lab, PT. Biham Riset dan Edukasi, Banda Aceh, 23243, Indonesia; d Medical Research Unit, School of Medicine, Universitas Syiah Kuala, Banda Aceh, 23111, Indonesia; e Department of Management, Institut Teknologi dan Bisnis Ahmad Dahlan Jakarta, Banten, 15419, Indonesia; f Tropical Disease Centre, School of Medicine, Universitas Syiah Kuala, Banda Aceh, 23111, Indonesia; g Department of Microbiology, School of Medicine, Universitas Syiah Kuala, Banda Aceh, 23111, Indonesia; h Department of Chemistry Education, Faculty of Education and Teacher Training, Universitas Syiah Kuala, Banda Aceh, 23111, Indonesia; i Department of Pharmacy, Faculty of Mathematics and Natural Sciences, Universitas Syiah Kuala, Banda Aceh, 23111, Indonesia; j Herbal Medicine Research Center, Universitas Syiah Kuala, Banda Aceh, 23111, Indonesia; k Department of Chemical Engineering, Faculty of Engineering, Universitas Malikussaleh, Aceh Utara, 24355, Indonesia; l Faculty of Public Health, Universitas Muhammadiyah Aceh, Banda Aceh, 23245, Indonesia

**Keywords:** Aromatherapy, Novel coronavirus, Pandemic, Research landscape, SARS-CoV-2

## Abstract

**Background:**

Essential oils are thought as potential therapies in managing coronavirus disease 2019 (COVID-19). Many researchers have put their efforts to tackle the pandemic by exploring antiviral candidates which consequently changes the research landscape. Herein, we aimed to assess the effect of COVID-19 pandemic toward the landscape of essential oil research.

**Methods:**

This study employed bibliometric analysis based on the metadata of published literature indexed in the Scopus database. The search was performed on December 15^th^, 2022 by using keyword ‘essential oil’ and its synonyms. We grouped the data based on publication year; pre-COVID-19 (2014–2019) and during COVID-19 (2020–2024, some studies have been published earlier). Further, we separated the COVID-19-focused research from COVID-19 (2020–2024) by introducing a new keyword ‘COVID-19’ during the search. All metadata were processed using VoSviewer and Biblioshiny for network visualization analysis. Selections of frequently occurring keywords, clusters of keyword co-occurrence, and the list of most impactful papers were performed by two independent reviewers.

**Results:**

Metadata from a total of 35,262 publications were included for bibliometric analysis, comprised of three groups of datasets namely pre-COVID-19 (*n* = 18,670), COVID-19 (*n* = 16,592), and COVID-19-focused (*n* = 281). Five research topics clusters were found from pre-COVID-19 dataset, eight – from COVID-19 dataset, and nine – from COVID-19-focused dataset. COVID-19 cluster containing the keyword ‘antiviral’ emerged in the COVID-19 dataset, whereas none of the previous research topic clusters contained the keyword ‘antiviral’. Antiviral, angiotensin-converting enzyme 2 (ACE2) inhibitory, and anti-inflammation activities were among the top occurring keywords in studies covering both essential oil and COVID-19. Studies on essential oil used for managing COVID-19 were most reported by authors from the United States (documents = 37, citations = 405), Australia (documents = 16, citations = 115) and Italy (documents = 23, citations = 366).

**Conclusion:**

A significant increase was found during COVID-19 pandemic for publications covering essential oil themes, but only a small portion was occupied by COVID-19 research. The COVID-19 pandemic does not alter the ongoing progress of essential oil research but rather offers a new spotlight on the antiviral potential of essential oils. Hence, the COVID-19 pandemic has provided an opportunity to investigate deeper the antiviral potential of essential oils.

## 1 Introduction

Coronavirus disease 2019 (COVID-19) has been the central of public health issue around the world since it was first declared as a pandemic in March 2020 [1]. The causative agent for this disease is a novel coronavirus, named severe acute respiratory syndrome coronavirus 2 (SARS-CoV-2) [2]. COVID-19 could cause a range of symptoms from flu-like symptoms (such as fever, cough, and sputum) to acute respiratory distress syndrome (ARDS) and death [3]. Systematic review and meta-analysis studies revealed that five most common symptoms of COVID-19 are fever, cough, fatigue, dyspnea, and sputum production [3,4]. Mortality among COVID-19 patients are caused by the disease progression into ARDS and multiple organ damages concomitant to the overwhelming and uncontrollable inflammatory response from the innate immune machinery [5]. As of January 11^th^, 2023, the number of death from COVID-19 pandemic has reached 6,708,763 worldwide with a total of 664.58 million infection cases [6].

In response to the ongoing pandemic, researchers have put their efforts in finding efficacious therapies with low risk of side effects. Multinational vaccination programs have shown a positive progress in preventing the transmission and hospital admission [7]. Monoclonal antibodies were found efficacious in providing early viral clearance and symptoms resolution [8]. However, emergence of new variants, especially B.1.1.529 (omicron), has set new challenges in curbing COVID-19 pandemic and its management [9,10]. More than 50 mutations in this variant, mostly in the spike protein, has allowed the virus to escape adaptive immunity, hence reduced efficacies of previous vaccines and monoclonal antibody therapies [11,12]. The present guideline suggests the use of remdesivir, nirmatrelvir, and molnupiravir in mild-to-moderate COVID-19 patients [13]. Remdesivir is not orally bioavailable and requires in-hospital setting for its injection administration [14]. Molnupiravir possesses genotoxicity and its prescription is limited to child-bearing and lactating individuals [15]. Moreover, data from real-world settings suggest lower efficacy of these drugs [16,17]. Therefore, the research on finding proper therapeutic strategies for COVID-19 should be continued.

Of complementary medicines used to treat COVID-19, essential oils have been suggested as a promising alternative [18,19]. Previously, studies have found that several essential oils are active against human immunodeficiency virus (HIV) [20] and SARS-CoV. Essential oils have been reported to ameliorate COVID-19-like symptoms including fever, cough, sputum, and fatigue [[21], [22], [23], [24]]. Thus, research on essential oils is potential to improve the COVID-19 management. Herein, we performed bibliometric evaluation on metadata of published essential oil-related literatures. By comparing the data before and during the COVID-19 pandemic, we are able to obtain the effect of the pandemic on the landscape of essential oil research. Bibliometric approach has been used previously to reveal the trend of research in medical fields [[25], [26], [27]]. But none has been used to reveal the impact of pandemic toward the research landscape, hence the novelty of the present study. The result of this study could inform researchers dealing with essential oil-related topics about research strategies and how to respond current research trend affected by COVID-19 pandemic.

## 2 Methods

### 2.1 Study design

This study was conducted to analyze the impact of COVID-19 pandemic on essential oils-related research by means of bibliometric analysis. Metadata of published literature were retrieved from Scopus database as of December 15^th^, 2022, comprising of papers reporting essential oils studies, where the datasets were assigned into three categories, namely pre-COVID-19, during COVID-19, and COVID-19-focused. A dataset from 2014 to 2019 were included in pre-COVID-19 group, while those from 2020 onward were included in during COVID-19 group. By applying additional keyword combinations, we obtained data from papers reporting both essential oils and COVID-19 data, where the dataset was labelled as COVID-19-focused. Vosviewer 1.6.17 (Centre for Science and Technology Studies, Leiden University, The Netherlands) and Biblioshiny [28], two most commonly utilized bibliometric software, were used to analyze the data through network visualization analysis.

### 2.2 Search strategy

Each dataset of pre-COVID-19, during COVID-19, and COVID-19-focused publications were collected from Scopus database. Datasets for pre-COVID-19 and during COVID-19 publications were filtered based on the publication year, where those published in 2014–2019 were assigned to pre-COVID-19 group and those published in 2020—onward were assigned to during COVID-19 group. Search terms used for the first two datasets were: “Aromatherapy” OR “Essential oil” OR “Aromatic plant”. COVID-19-focused dataset was retrieved by documents published in 2020—onward, refined by publication year filter. The keywords combination used for COVID-19-focused dataset was as follow: (“Aromatherapy” OR “Essential Oil” OR “aromatic Plant”) AND (“COVID” OR “SARS-CoV-2” OR “Coronavirus*“); in which the combination was aimed to ensure the data were solely focusing on COVID-19 related studies. The searches were executed on the title, abstract, or keywords of the published literature. The limitation and exclusion of the literature searches are presented in flowcharts (Figs. S1, S2, and S3). Specific subject areas were excluded in this literature search to ensure the relevancy of essential oils and COVID-19 related fields, they were: ‘Arts & Humanities’, ‘Economics, Econometrics & Finance, Business, Management & Accounting’, ‘Social Sciences’, ‘Earth & Planetary Sciences’, ‘Computer Sciences’, and ‘Energy’. All retracted and erratum documents and non-English papers were excluded. The information of authors, title, abstract, author's affiliation, author and journal's keyword and journal's title were extracted and exported as CSV file (.csv).

### 2.3 Network visualization and keyword analysis

Network visualization analysis was conducted using VosViewer by inputting the CSV (.csv) file retrieved from Scopus database. The analysis generated a mapping for each file in accordance with the clusters of linked author keywords, where linked keywords were classified under the same colour. Exclusions were made on clusters containing overlapping keywords or non-meaningful keyword combinations. Each keyword in the mapping was represented in a node, which size was determined by the keyword's link strength or how many other keywords were linked to it.

The most frequent bioactivities or functions of essential oils were initially selected based on their number of occurrences. Two authors (B.G. and M.I.) further performed the selection to avoid the overlapping of keywords, as two different keywords could have a single meaning. For instance, ‘antibacterial’ and ‘antimicrobial’ were considered overlapping keywords and ‘antimicrobial’ was selected as the representative.

### 2.4 Selection of the most cited paper

The number of citations was used as a parameter of the scientific report's impact. The articles were firstly sorted by their number of citations. The selection was continued by the authors (B.G. and M.I.) by performing full-text screening to ensure that each article report had a sufficiently significant information about ‘essential oils’ and ‘COVID-19’. Papers that only slightly mentioned ‘essential oils’ and ‘COVID-19’ and their synonyms were excluded from the list.

## 3 Results

### 3.1 Number and types of publications

**Fig. 1:**
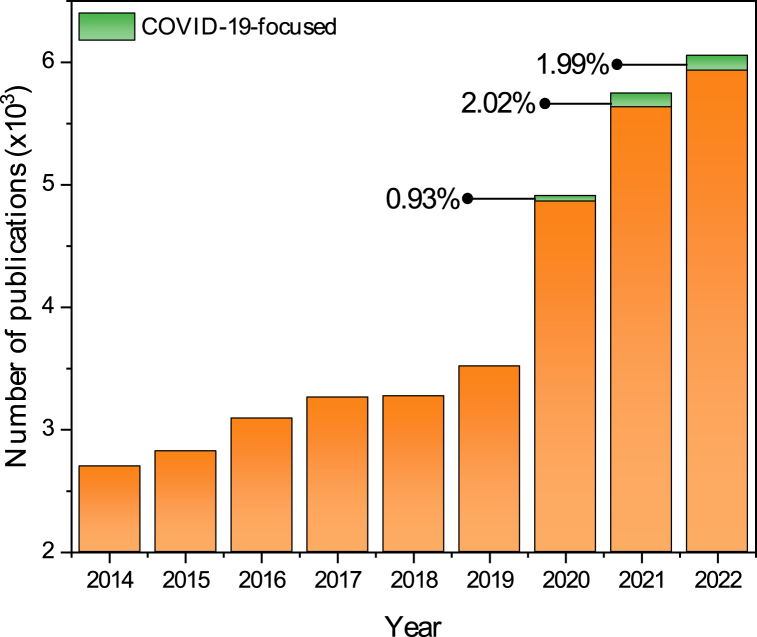
Annual number of publications covering essential oil-related topics and those with COVID-19 focused studies.

A total of 35,262 papers were retrieved from the three datasets (essential oils pre-COVID-19 (*n* = 18,670), essential oils during COVID-19 (*n* = 16,592), essential oils and COVID-19 (*n* = 281)), which consisted of original research articles (*n* = 30,375; 86.14%), review articles (*n* = 3252; 9.22%) and other document types including conference paper, book chapter, editorial and letter (*n* = 1635; 4.63%). The document types of each dataset are presented in Table 1. Number of annual publications along with the proportion of COVID-19-focused research are presented in Fig. 1.

**Table 1:** Document type of essential oils research (2014–2024).

Document type	Number of papers (%)		
	Pre-COVID-19 (*n* = 18670)	During COVID-19 (*n* = 16592)	COVID-19 Focused (*n* = 281)
Original Research	16535 (88.56%)	13840 (83.41%)	159 (56.59%)
Review	1256 (6.73%)	1996 (12.03%)	94 (33.45%)
Book chapter	462 (2.47%)	379 (2.29%)	6 (2.13%)
Conference paper	169 (0.91%)	185 (1.11%)	0 (0%)
Note	78 (0.42%)	25 (0.15%)	0 (0%)
Letter	57 (0.31%)	48 (0.29%)	6 (2.13%)
Editorial	56 (0.3%)	75 (0.45%)	13 (4.63%)
Short survey	30 (0.16%)	30 (0.18%)	2 (0.71%)
Book	27 (0.14%)	14 (0.09%)	1 (0.36%)

Pre-COVID-19: 2014–2019; COVID-19: 2020–2024; COVID-19-focused: 2020–2024.

The publication on essential oils research increased by almost 40% once COVID-19 pandemic began (3517 and 4865 papers in 2019 and 2020, respectively). However, the number of COVID-19 focused research on essential oils were considered inferior to the rest of the papers, as there were only 281 papers (1.69%) described essential oil and COVID-19. Despite its sheer number, the annual increase of the essential oils research focusing on COVID-19 could be observed.

### 3.2 Bioactivities and functions of essential oils based on keyword occurrence frequency

**Fig. 2:**
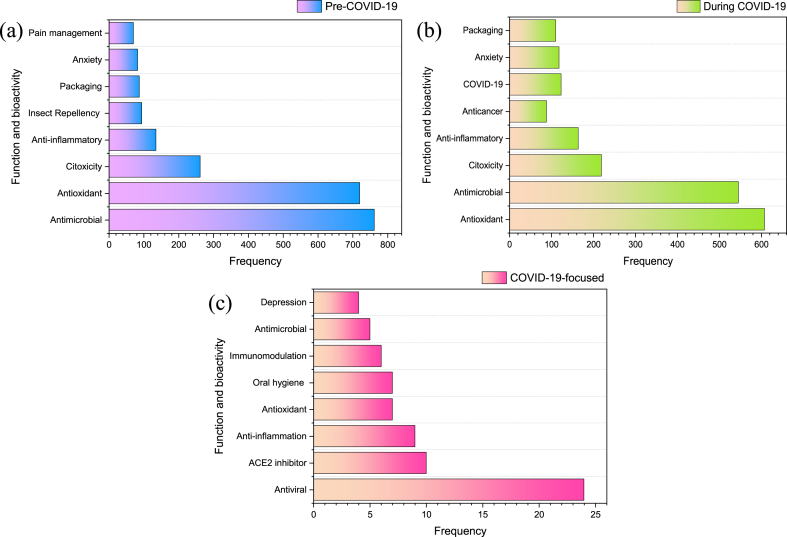
The most frequently reported bioactivities and functions of essential oils based on the keyword occurrence.

The most commonly studied bioactivities and functions of essential oils as reflected by the keyword occurrences are presented in Fig. 2. Antimicrobial, antioxidant, cytotoxicity, and anti-inflammatory activities of essential oils remained the top four bioactivities reported in pre- and during COVID-19 era. Investigations of essential oils for insect repellency and pain management functions were no longer frequent after 2020, where there were new keywords emerged including ‘anticancer’, ‘COVID-19’, and ‘packaging’. Dataset from COVID-19-focused essential oil research revealed that antiviral, ACE2 inhibition, anti-inflammation, and antioxidant were among the most frequently studied bioactivities (Fig. 2). Essential oils as oral hygiene product was among the most mentioned keyword from the dataset of COVID-19 focused research.

### 3.3 Co-occurrence of all keywords

**Fig. 3:**
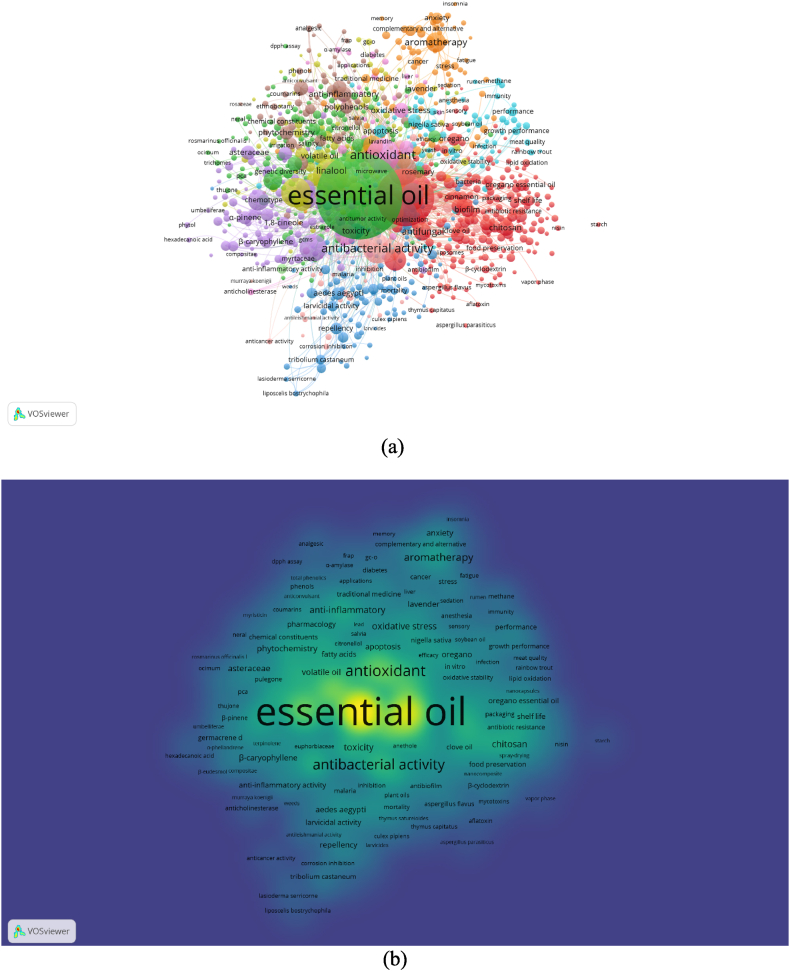
(a) Network visualization of all keywords (weights: occurrences). (b) Overlay visualization of all keywords in 2014–2019 (weights: occurrences, score: average publications per year). Nodes with the same color represent a research topic cluster, where the red color is for food packaging; navy blue for insecticidal, larvicidal and fumigant; yellow for separation and identification methods of essential oils; orange for pain, stress and depression management; and brown for anti-inflammatory and antimicrobial agents. (For interpretation of the references to color in this figure legend, the reader is referred to the Web version of this article.)

**Fig. 4:**
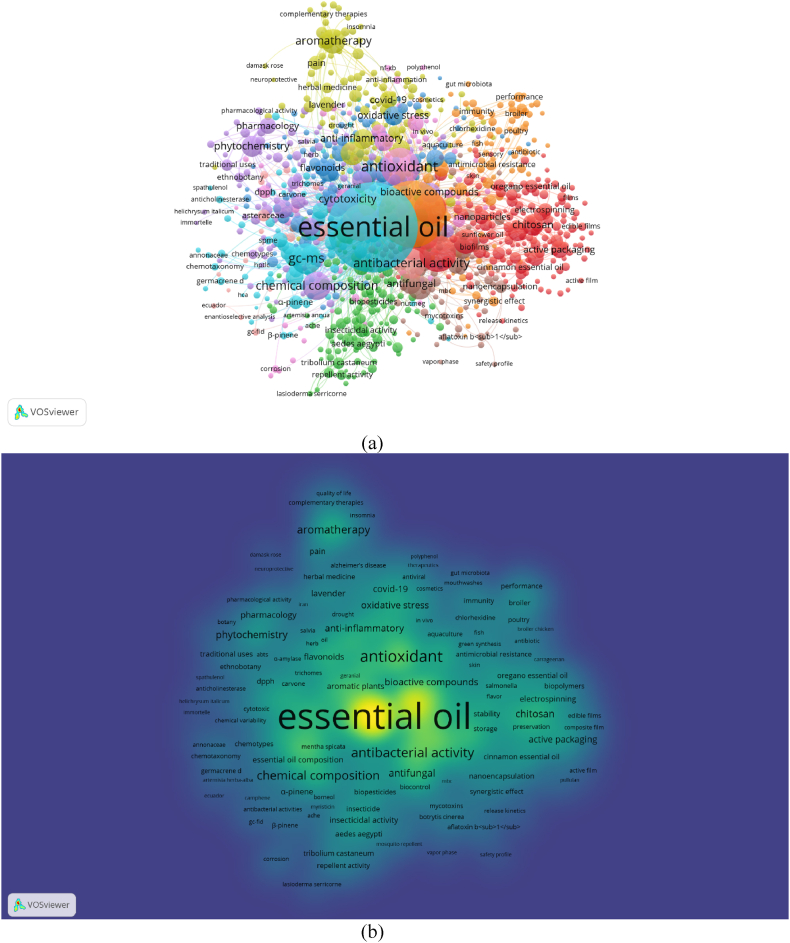
(a) Network visualization of all keywords (weights: occurrences). (b) Overlay visualization of all keywords in 2019–2022 (weights: occurrences, score: average publications per year). Nodes with the same color represent a research topic cluster, where the red color for food packaging; green for insecticidal, insect repellent, larvicidal, and fumigant; navy blue for antioxidant activities; avocado green for essential oils in COVID-19 management and their antiviral activities; purple for anticancer agents; cyan for separation and identification methods for essential oils; orange for poultry; dark red for antimicrobial activities of essential oils. (For interpretation of the references to color in this figure legend, the reader is referred to the Web version of this article.)

**Fig. 5:**
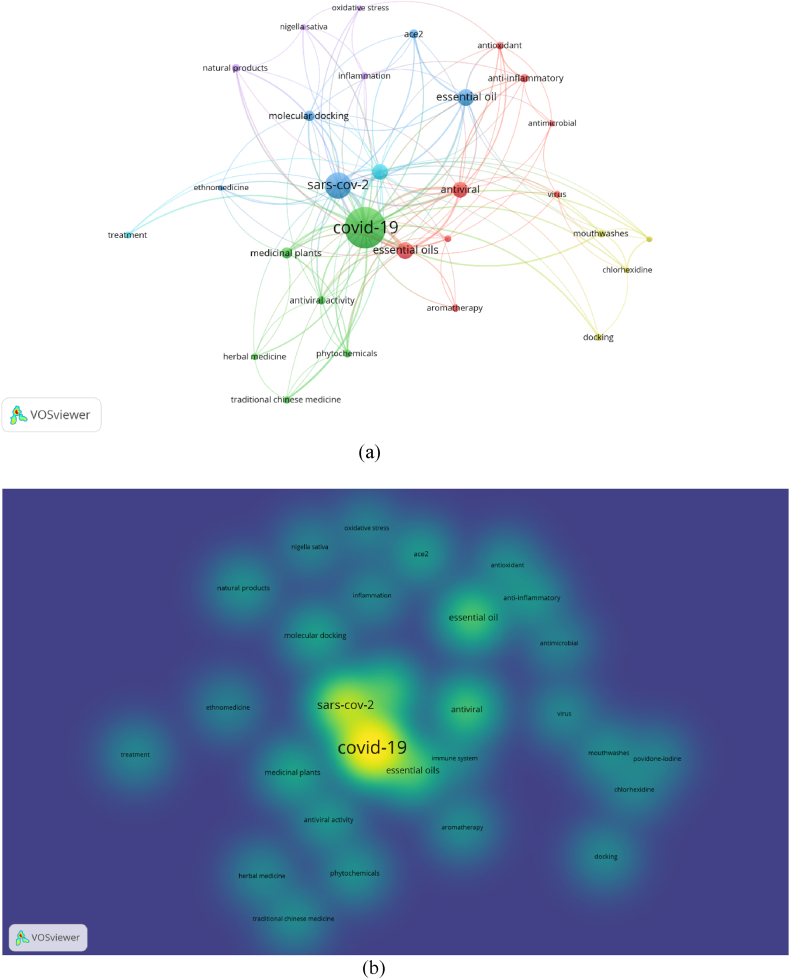
(a) Network visualization of all keywords (weights: occurrences). (b) Overlay visualization of all keywords for COVID-19 focused essential oils research (weights: occurrences, score: average publications per year). Nodes with the same color represent a research topic cluster, where the green color for essential oils as immunomodulator against SARS-CoV-2; red for essential oils as antioxidant, anti-inflammatory and anti-microbial agents; in blue for computational approach in predicting antiviral activities of essential oils; and yellow for mouth rinsing in managing COVID-19. (For interpretation of the references to color in this figure legend, the reader is referred to the Web version of this article.)

The network visualizations of all keywords co-occurrence are presented in Fig. 3, Fig. 4, Fig. 5. For the purpose of having a better analysis on the keywords' co-occurrences, restriction was enacted on essential oils research in pre-COVID-19 (2014–2019) and during COVID-19 (2020–2022), where the minimum number of occurrences of a keyword was set to 5, while COVID-19 focused essential oils research's minimum number of occurrences of a keyword was set to 3. The latter received lower number of minimum number of occurrences of a keyword due to the significantly lower number of keywords available in the metadata (*n* = 922), compared to the other two metadata in pre-COVID-19 (*n* = 30999) and during COVID-19 (*n* = 30664).

During pre-COVID-19 (2014–2019), 2568 keywords met the threshold and the 1000 keywords with the greatest total link strength were selected to feature in the mappings (Fig. 3a). The most used main keyword in essential oils research during pre-COVID-19 was ‘essential oil’ or ‘essential oils’ (occurrences = 6377), followed by ‘antimicrobial activity’ (occurrences = 762), ‘antioxidant activity’ (occurrences = 720), ‘antioxidant’ (occurrences = 646), ‘GC-MS’ (occurrences = 530) and ‘antibacterial activity’ (occurrences = 507). These six keywords were the only keywords to occur in essential oils research pre-COVID-19 for more than 500 times. The colors shown in the network visualization presented the closeness of the keywords to a certain research topic. The five largest research topics formed clusters which were comprised of keywords related to ‘food packaging, insecticidal, separation and identification methods’, ‘pain and stress management’, and ‘anti-inflammatory and antimicrobial activities’ (Fig. 3a).

The density visualization shows how the study mainly revolved around certain keyword(s). For the pre-COVID-19 dataset the density visualization is presented in Fig. 3b. Keyword in yellow occurred in papers with the highest density, followed by light green to darker green. The mapping suggested that essential oil was the keyword with highest density, while a small portion of keywords were also found revolving around ‘antioxidant activity’, ‘aromatherapy’, and ‘chitosan’.

During COVID-19 (2020–222), 2340 keywords met the threshold while top 1000 keywords according to its total link strength were featured in the mappings (Fig. 4a). There were no significant changes to the most used keywords, as ‘essential oil/essential oils’ (occurrences = 5121), followed by ‘antioxidant’ (occurrences = 608), ‘antioxidant activity’ (occurrences = 554), ‘antimicrobial activity’ (occurrences = 546), all five which made it through the 500-occurrences mark. Most frequently studied topics found in the clusters are ‘food packaging’, followed by clusters comprised of ‘insecticidal, insect repellent, larvicidal, and fumigant’ keywords. There are other six major clusters formed by keywords related to antioxidant activities, COVID-19, anticancer, separation and identification methods, poultry, and antimicrobial activities (Fig. 4a).

Based on the density visualization, keyword in yellow occurred in papers with the highest density, followed by light green to darker green (Fig. 4b). Same result as the previous mapping was obtained, where ‘essential oil’ was the keyword with highest density, although several keywords' density is considered larger than the rest, including ‘antimicrobial activity’, ‘GC-MS’ and ‘antioxidant’ (Fig. 4b).

As for COVID-19 focused research on essential oils, of the 922 identified keywords, only 29 met the threshold and thus were shown in the mappings (Fig. 5a). A change occurred among the most used keywords, where ‘COVID-19’ (occurrences = 120) topped all other keywords, followed by ‘SARS-CoV-2’ (occurrences = 56), ‘essential oils’ (occurrences = 54), ‘coronavirus’ (occurrences = 24) and ‘antiviral’ (occurrences = 24). Only one keyword passed the 100 occurrences mark, which is deemed acceptable due to the publication recency and relatively new research field. The colors shown in the network visualization presented the closeness of the keywords to a certain research topic. The largest research topics cluster formed by studies reporting essential oils as immunomodulator against SARS-CoV-2. The second largest research topics cluster is comprised by keywords related to anti-inflammatory and anti-microbial activities. ‘*Anti*-SARS-CoV-2’ cluster places the third position, where keywords related to computational methods in probing the antiviral activities are contained in the cluster (Fig. 5a).

The density visualization shows that keyword in yellow occurred in papers with the highest density, followed by light green to darker green (Fig. 5b). COVID-19 and SARS-CoV-2 were the keywords with highest densities, with all other keywords revolving around the aforementioned keywords (Fig. 5b).

### 3.4 Clusters from the network visualization of keyword co-occurrence

Clusters and suggested research hotspots identified from the network visualization of keyword co-occurrence are presented in Table 2. As many as five clusters were identified from the network visualization during the period of 2014–2019 (pre-COVID-19). The number of clusters increased to eight once the research entered 2020 (during COVID-19). The main cluster found in pre-COVID-19 group suggested the research of hotspot ‘utilization of essential oil in food packaging’, which remained the same in COVID-19 period. Similar to the application of essential oils as insecticidal or fumigant, especially against *Aedes aegypti*, in which this research hotspot appeared second in both pre- and during COVID-19 periods. ‘Antiviral’ cluster suggesting the application of essential oil in COVID-19 management newly emerged during the pandemic. It is worth noting that emergence of new clusters within the time span of 2020–2024 were also found from studies investigating essential oil utilization as bird supplement and anticancer.

**Table 2:** Clusters formed by keyword co-occurrence indicating the research hotspots.

Cluster	Comprising keywords	Research hotspot(s)
Pre-COVID-19		
I	Essential oils, antifungal activity, antibiotics, edible film, chitosan, encapsulation, food safety, active packaging, biofilm, and nanoemulsion	Utilization of essential oil in food packaging
II	Toxicity, repellency, *Aedes aegypti*, insecticidal activity, larvicidal activity, fumigant toxicity, repellent, phytotoxicity, botanical insecticides, acute toxicity	Essential oils as insecticidal, larvicidal, and fumigant
III	Antioxidant activity, GC-MS, hydrodistillation, super critical fluid extraction, extraction, oil, anticholinesterase activity, volatile oil, HPLC, and oxidative stability	Separation and identification methods for essential oils
IV	Medicinal plants, lavender, natural products, aromatherapy, anxiety, pain, massage, stress, traditional medicine, and depression	Essential oil as pain, stress, and depression management
V	Antimicrobial, flavonoids, anti-inflammatory, polyphenols, DPPH, pharmacology, phytochemistry, biological activity, and terpenoids	Essential oil as antioxidant, anti-inflammatory, and antimicrobial agents
During COVID-19		
I	Antimicrobial activity, nanoemulsion, chitosan, encapsulation, carvacrol, stability, active packaging, edible films, food safety, thymol	Utilization of essential oil in food packaging
II	Toxicity, gas chromatography, insecticidal activity, repellency, larvicidal activity, integrated pest management, fumigant toxicity, botanical insecticides, eucalyptus, fungi	Essential oils as insecticidal, insect repellent, larvicidal, and fumigant
III	Flavonoids, secondary metabolites, phenolic compounds, antioxidants, oxidative stress, linalool, polyphenols	Antioxidant activities of essential oil
IV	Aromatherapy, COVID-19, anti-inflammatory, medicinal plants, pain, molecular docking, phytochemicals, lavender, antiviral, herbal medicine	Essential oils in COVID-19 management and their antiviral activities
V	Chemical composisiton, terpenes, lamiaceae, biological activities, phytochemistry, pharmacology, pharmacokinetics, antiproliferative, cytotoxic, bioactive compounds	Essential oils as anticancer agents
VI	Essential oil, GC-MS OR GS/MS, hydrodistillation, essentail oil compounds, volatile oil, α-pinene, myrtaceae, LC-MS, metabolomics, and extraction	Separation and identification methods for essential oils
VII	Essential oils, oregano, growth performance, poultry, immunity, gut health, thyme, plant extract, bacteria, and antimicrobial resistance	Utilization of essential oil in poultry
VIII	Antibacterial, antifungal, antifungal activity, biofilm, *Staphylococcus aureus*, eugenol, mycotoxins, nanoencapsulation, synergism, and *Candida albicans*	Antimicrobial activities of essential oils
COVID-19-focused		
I	Anti-inflammatory, antibacterial, antimicrobial, antioxidant, antiviral, carvacrol, complementary medicine, phytomedicine, thymoquinone, essential oil	Essential oil as antioxidant, anti-inflammatory, and antimicrobial agents
II	2019-nCoV, immunity, medicinal plants, phytochemicals, SARS-CoV, traditional Chinese medicine, traditional medicine, viral infections	Essential oils as immunomodulator against SARS-CoV-2
III	Anxiety, aromatherapy, depression, mental health, and stress	Essential oils in managing depression and anxiety associated with COVID-19
IV	Antiviral activity, bioactive compounds, COVID-19, docking, eucalyptus oil, herbal medicine, natural product	Antiviral activities of essential oils
V	Inflammation, natural compounds, natural products, nigella sativa, oxidative stress, pandemic, and SARS-CoV-2	Essential oils in controlling inflammatory response associated with SARS-CoV-2 infection
VI	Chlorhexidine, mouthwashes, povidone-iodine, viral load, virucidal activity, and virus	Essential oils for mouth rinsing in managing COVID-19
VII	Essential oils, GC-MS, in silico, molecular docking, terpenes, therapeutics	The use of computational approach in predicting antiviral activities of essential oils
VIII	Anosmia, olfactory training, SARS-CoV-2	Essential oil usage in managing COVID-19 neuro symptoms
IX	ACE2, eucalyptus	Essential oil as ACE2 inhibitor

Note: Clusters are ranked based on the size.

Datasets from publications covering both essential oil and COVID-19 topics were found to be comprised of 9 distinct research hotspots. Essential oils properties of being antioxidant, anti-inflammatory, and antimicrobial emerged as the primary cluster. Essential oils to modulate immune response during the course of SARS-CoV-2 infection were the second most studied topic, followed by the potential of essential oils in the management of COVID-19 impacts, especially those associated with depression and anxiety. The antiviral activities of essential oils were also among the research hotspots which occupied the fourth position.

### 3.5 Co-authorship countries

**Fig. 6:**
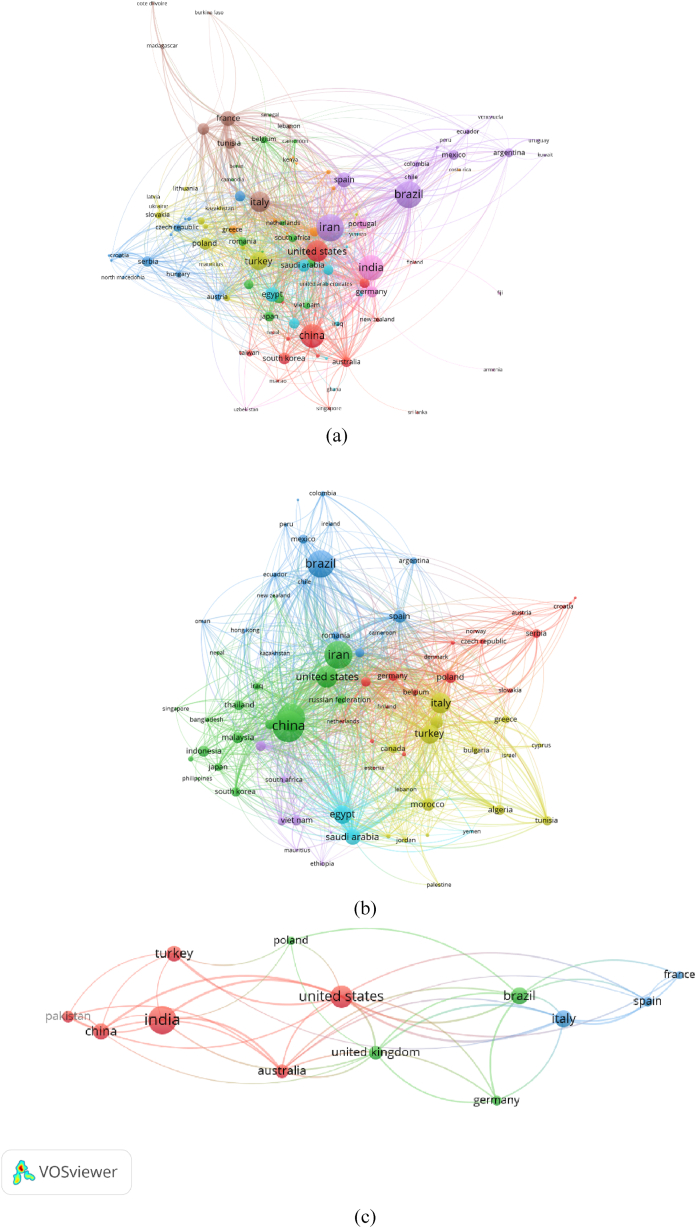
Network visualizations of co-authorship countries for essential oil-related research pre-COVID-19 (a), during COVID-19 (b), and COVID-19-focused (c).

The network visualization and overlay visualization maps of the co-authorship countries on the related studies are presented in Fig. 6, each representing the mapping of essential oils research pre-COVID-19, during COVID-19 and COVID-19 focused study, respectively. Restriction was conducted to assess the countries' co-authorship, such as a maximum of 25 countries per document, a minimum of five documents of a country, and limiting the number of citations of a country to 100. A network visualization and density visualization were then presented to interpret the countries’ co-authorship and collaboration of each identified groups of countries.

A total of 202 countries published essential oils papers during pre-COVID-19 and only 107 met the thresholds after the restriction criteria were applied. The top three countries were United States (documents = 1384, citations = 31799, TLS = 1143), Italy (documents = 1211, citations = 32916, TLS = 987) and France (documents = 658, citations = 13680, TLS = 634). Interestingly, the three countries did not publish the highest number of papers, as Iran (Documents = 2225, citations = 43482, TLS = 622), Brazil (Documents = 2160, citations = 40976, TLS = 544) and India (Documents = 1880, citations = 32119, TLS = 527) were all placed above despite having lower total link strength. Egypt and Saudi Arabia had the most collaborations (LS = 153), followed by China and United States (LS = 129) and United States and Brazil (LS = 94).

There was an increase in number of countries publishing essential oils research during COVID-19 period (2020-), where 237 countries were identified. Of these countries, 80 met the thresholds. United States (Documents = 1124, citations = 6761, TLS = 1140), Saudi Arabia (Documents = 689, citations = 4403, TLS = 940) and Italy (Documents = 1047, citations = 8340, TLS = 940) were the top three countries. Same situation to the previous countries’ co-authorship result, the three countries did not publish the highest number of papers, as China (Documents = 2171, citations = 14596, TLS = 794), Iran (Documents = 1789, citations = 11095, TLS = 852) and India (Documents = 1786, citations = 8527, TLS = 828) topped the highest number of papers published, again placed below the top three countries due to their lower total link strength. Egypt and Saudi Arabia also had the most collaborations (LS = 319), followed by United States and China (LS = 143) and United States and Brazil (LS = 101).

As for essential oils research on COVID-19, 71 countries were identified and only 13 met the thresholds. Of these countries, United States (Documents = 37, citations = 405, TLS = 32), Australia (Documents = 16, citations = 115, TLS = 19) and Italy (Documents = 23, citations = 366, TLS = 19) were the top three countries. India (Documents = 55, citations = 369, TLS = 15) published the highest number of papers. Four collaborations: China and United States, Italy and United States, India and United States and India and Australia all had the most collaborations (LS = 4), although the number of collaborations were still considered majorly inferior when compared to overall essential oils research.

**Fig. 7:**
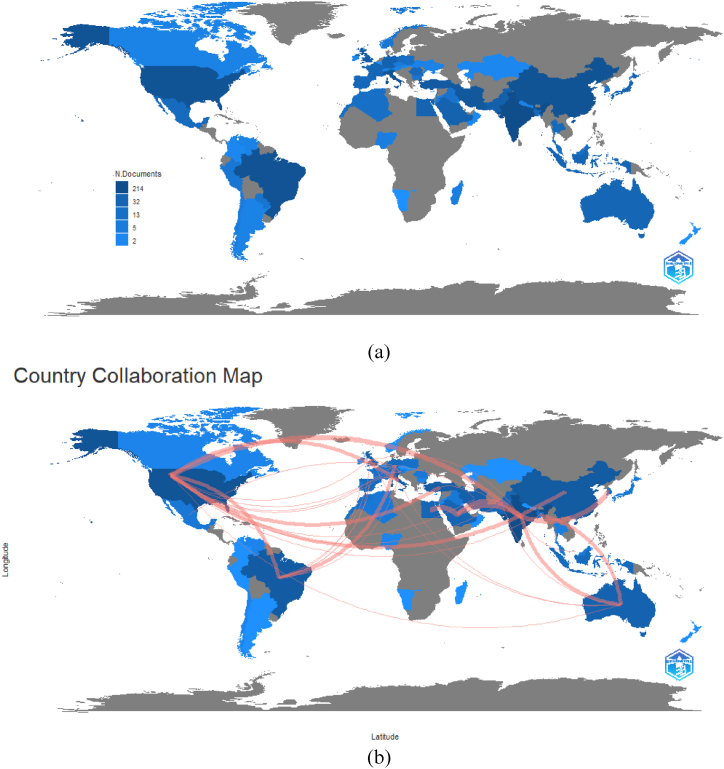
Country scientific production (a) and collaboration map (b) of studies covering both essential oils and COVID-19 topics (2020–2022).

Further mappings were conducted using Biblioshiny to identify the country collaboration and country scientific production of essential oils research on COVID-19, where the generated map has been presented in Fig. 7. Similar to the VosViewer mappings, United States, Brazil, India and China were the most productive countries. In terms of collaboration, United States and Australia collaborated with the highest number of countries, as seen in both VosViewer's network visualization and Biblioshiny's country collaboration map, which satisfy their stance as two of the four countries who had the highest number of collaborations.

### 3.6 Most cited literatures covering essential oil and COVID-19 topics

The list of top cited papers reporting essential oil used in COVID-19 management has been presented in Table 3. The first on the list was a paper entitled “Investigation into SARS-CoV-2 resistance of compounds in garlic essential oil” authored by Bui Thi Phuong Thuy and colleagues receiving 152 citations. The second position was occupied by a review article “COVID-19: Is there evidence for the use of herbal medicines as adjuvant symptomatic therapy?” authored by Dâmaris Silveira and colleagues with citations number reaching 113. Other than these two articles, all studies had citations of less than 100. In comparison, we also performed the analysis on essential oil-themed papers published during pre-COVID-19 (Table S1) and during COVID-19 (Table S2). All papers in the lists reached over 100 citations, even passing 400 citations milestone for papers published in 2014–2019. The highest number of citations achieved by papers published in pre-COVID-19 was 699 (Table S1). Meanwhile, 235 citations was the highest number of citations achieved by studies published during COVID-19 (Table S2).

**Table 3:** Top 10 most cited papers covering the topics of essential oils and COVID-19.

Rank	Title	Type	Year [Ref]	Times of Citations	First author		Lead author*	
					Name	First year reporting essential oil	Name	First year reporting essential oil
1	Investigation into SARS-CoV-2 resistance of compounds in garlic essential oil	Original research	2020 [29]	152	Bui Thi Phuong Thuy	2020	Duong Tuan Quang	2020
2	COVID-19: Is there evidence for the use of herbal medicines as adjuvant symptomatic therapy?	Review	2020 [30]	113	Dâmaris Silveira^a^	1996	Jose Maria Prieto-Garcia	2001
							Michael Heinrich	1996
3	COVID-19 and therapy with essential oils having antiviral, anti-inflammatory, and immunomodulatory properties	Review	2020 [31]	96	Muhammad Asif^a^	2020	–	–
4	In silico study the inhibition of angiotensin converting enzyme 2 receptor of COVID-19 by *Ammoides verticillata* components harvested from Western Algeria	Original research	2021 [32]	94	Imane Abdelli	2021	Fayçal Hassani	2008
5	Computational evaluation of major components from plant essential oils as potent inhibitors of SARS-CoV-2 spike protein	Original research	2020 [33]	76	Seema A. Kulkarni	2020	Periyar S. Sellamuthu	2013
							Thirumurthy Madhavan	2020
6	Aromatic herbs, medicinal plant-derived essential oils, and phytochemical extracts as potential therapies for coronaviruses: Future perspectives	Review	2020 [34]	57	Mohamed N. Boukhatem^a^	2011	–	–
7	Geranium and lemon essential oils and their active compounds downregulate angiotensin-converting enzyme 2 (ACE2), a SARS-CoV-2 spike receptor-binding domain, in epithelial cells	Original research	2020 [35]	56	K. J. Senthil Kumar	2020	Sheng-Yang Wang	2001
8	An updated and comprehensive review of the antiviral potential of essential oils and their chemical constituents with special focus on their mechanism of action against various influenza and coronaviruses	Review	2021 [36]	55	Abdul Rouf Wani	2021	Kanchan Yadav	2021
							Manzoor Ahmad Rather	2011
9	Challenges at the time of COVID-19: opportunities and innovations in antivirals from nature	Review	2020 [37]	50	Andreas Hensel^a^	2007	–	–
10	Chinese herbal medicine: Fighting SARS-CoV-2 infection on all fronts	Review	2021 [38]	43	Zhonglei Wang^a^	2021	Liyan Yang	2021

*Lead authors are indicated by their role as corresponding authors

a This author also acted as corresponding author

The number of citations of essential oils research during COVID-19 (2020–2022) was considerably lower, where Homaeigohar and Boccaccini paper “Antibacterial biohybrid nanofibers for wound dressings” in 2020 was cited in only 235 papers. The second and third rank were no difference, where “Chemical constituents and pharmacological activities of garlic (*Allium sativum* L.): A review” authored by Batiha and colleagues and “Chitosan nanoparticles loaded with clove essential oil: Characterization, antioxidant and antibacterial activities” authored by Hadidi and colleagues were cited for 212 and 198 times, respectively. It is interesting to note, however, there was one COVID-19 focused paper ranked in the top 10 most cited papers during COVID-19, in which Thuy and colleagues’ paper “Investigation into SARS-CoV-2 resistance of compounds in garlic essential oil” in 2020 was cited in 152 papers.

## 4 Discussion

To report the trend of essential oils research across the years in pre- and during COVID-19, a bibliometric analysis was conducted to a total of 35,262 identified papers retrieved from Scopus database. The trend of essential oils research was considered everchanging, as the number of research hotspots were increasing over the years, especially during the transition from pre- and during COVID-19 pandemic. The leading research hotspots remained the same, as food packaging and insecticidal, larvicidal and fumigant were the most pursued research fields. The new research hotspots appearing during COVID-19 were also raising from entirely new keywords which were not emerged in pre-COVID-19, one of them being essential oils utilization in poultry. Other research hotspots were raised from a combination of keywords of multiple research hotspots during pre-COVID-19, including essential oils in COVID-19 management and their antiviral activities, which contained a combination of aromatherapy and anti-inflammatory, comprising keywords from essential oils as ‘pain, stress, and depression management’ and ‘essential oils as antioxidant, anti-inflammatory, and antimicrobial activities’.

In terms of the citation number studies reporting ‘essential oils for COVID-19’ was still considered inferior (with the most cited paper in this topic only received 100 citations [29]. The same case occurred in review article publications, as only one paper was cited for more than 100 times, highlighting the application of a wide range of herbal medicines, including essential oils, as potential adjuvants to treat COVID-19 [30]. The inferiority of essential oils research on COVID-19 is considered understandable, baring its relatively new field of study, which was not around until the start of 2020, thus showing an opportunity to investigate the antiviral potential of essential oils deeper for a better preparation for future pandemic.

### 4.1 Changes in essential oil research trends during COVID-19 pandemic

#### 4.1.1 Evidenced by number of publications

Annual number of publications of essential oil research, regardless the document type, increased dramatically in 2020. The number was maintained in increasing trend in the following years. This finding is interesting because COVID-19 was firstly declared in early 2020, where many research facilities experienced a lockdown, and consequently hindered researchers to perform the experiment [39]. There were indeed differences of document type percentages, where the percentage of review paper was observed higher during COVID-19 pandemic than pre-COVID-19 (12.03% versus 6.73%). At the same time, percentage of ‘original research documents reduced by 5.15% during the COVID-19 pandemic. This suggests that researchers maintain their writing productivity by converting the laboratory-based research activities to desk research activities. This is similar to the percentages of ‘editorial’ documents, in which the percentage was observed higher during the COVID-19 compared to pre-COVID-19 (0.16% versus 0.18%). A review article has suggested that desk research and secondary data evaluation could be the alternative for researchers amidst the lockdown [40]. Though it is true that researchers might prepare their unpublished paper during this period, but desk research activities remained significant (as higher publication number is observed in review document). However, in our previous work, we found a rather sluggish progress of laccase-assisted wastewater treatment research in 2020, but it rapidly returned to increasing trend in 2021 [41]. Further research could be carried out to comprehend the adaptive ability of researchers during the pandemic.

More interesting finding was found on ‘conference paper’, where higher percentage was recorded during COVID-19 compared with pre-COVID-19 (*n* = 169; 0.91% versus *n* = 185; 1.11%). Amidst COVID-19 pandemic, the travel bans and mass gathering restriction were imposed which essentially hindered the conference to be held. However, the options of conducting scientific conference through online platform allow researchers to participate the conference remotely. The shift from offline conference to online conference may incentivize researchers to participate as they are not required to make additional spending on flight tickets and hotel accommodation.

Types of paper covering both essential oil and COVID-19 topics are still dominated by original research by 56.59%. However, this number is relatively lower as compared to the proportion of document types published before and during the COVID-19 pandemic. Consequently, review articles and editorials occupied higher proportion (33.45% and 4.63%, respectively). During the early time of pandemic, there are difficulties in investigating the potentials of essential oils to treat COVID-19, including the availability of proper and widely available in vitro or in vivo models [42]. Even today, researchers are still developing proper experimental models to study *anti*-SARS-CoV-2 candidates. This limitation might contribute to the low percentage of essential oil research for COVID-19 management, where only 45 (0.93%), 114 (2.02%), and 122 (1.99%) papers published in 2020, 2021, and 2022, respectively. Moreover, despite the growing number of published papers covering both essential oil and COVID-19 topics (from 114 to 122), its proportion turned to be slightly lower (from 2.02% to 1.99%). This is indicative that researchers who have dealt with essential oils topic prior to the pandemic are unlikely to change their research focus to COVID-19. Apart from the limited availability of experimental protocols to study *anti*-SARS-CoV-2 activity, researchers might face difficulties to switch into a new topic as they are required to acquire new knowledge and research facilities (such as the requirement to perform the experiment in a biosafety level-3 facility). Additionally, research carrying this topic might have been performed but have not been published.

#### 4.1.2 Evidenced by research hotspots

In network visualization analysis, research topics that have been well established would form their respective cluster of keywords and can be translated as research hotspots. The analysis revealed that research hotspots of investigating essential oils applications in food packaging, controlling the population of disease bearing mosquitos and management of mental illness. The keyword co-occurrence also formed two other clusters for extraction and identification of essential oils and their bioactivities including antioxidant, anti-inflammatory, and anti-microbial activities. Based on 2020–2024 publication data, essential oils application as food packaging bioadditives and fumigants remained as major research hotspot. During this time frame, antioxidant and antimicrobial activities of essential oils formed their respective cluster, where larger cluster was observed for the former. Most importantly, we notice the formation of cluster attributed to COVID-19 management indicating the rapid increase of this research focus. This is also the first time that essential oil research received a spotlight for antiviral activities. Moreover, the formation of anti-inflammation as a single research hotspot might be attributed to the formation of ‘COVID-19’ cluster. Bioactivities of essential oils that are beneficial for COVID-19 management, including the *anti*-SARS-CoV-2 activities, was found as the frequent keyword. Two crucial research topics, ‘anticancer’ and ‘poultry’, emerged as research hotspots during COVID-19 pandemic. This is corroborated by keyword occurrence frequency data, where anticancer activities are frequently reported during that period of time. This suggests that COVID-19 pandemic does not interfere the trend development of essential oil research. Taken altogether, the most significant effect of COVID-19 pandemic toward the essential oil research landscape is the new research spotlight on antiviral potential of essential oils.

#### 4.1.3 Evidenced by researcher profiles

The collaboration network analysis among countries suggests a rather dynamic trend in this research topic. Collaboration between authors from Brazil and Iran was firstly the main contribution to essential oil research. USA and China have established a strong collaboration, with China dominating the Asia Pacific region. India formed partnerships with European countries such as Portugal and Germany in reporting essential oils. Meanwhile, Turkey had a strong partnership with Poland connecting the country with other Northern European countries (Latvia, Ukraine, and Slovakia).

After 2020, the collaboration expanded and more intense for Iran, China, and United States network. China remained as the leading country for Asia region. Saudi Arabia and Egypt was found to form its own consortium, indicating the increasing number of contributors from Middle East and North Africa (MENA) regions. Research interest on essential oil also grew among South American countries with Brazil as the leading country. Similar to European countries, where Poland was revealed to be the leader in this research topic. These findings are evident that COVID-19 pandemic does not hinder the proliferation growth of essential oil research.

By comparing the data with the collaboration network occurred for research papers covering both essential oils and COVID-19, we may observe that despite its limited number, the collaboration is more international. For instance, the major contributor for the topic is India forming collaboration with United States, Turkey, China, Pakistan, and Australia. Brazil on the other hand dominated the other cluster, comprised of countries from European continent (United Kingdom, Poland, and Germany). Still, a research consortium among the same European countries (France, Spain, and Italy) could be observed. These findings are confirmed by the collaboration map, in which despite a few countries contributing to the research topic (essential oils for COVID-19 management), the collaboration was intercontinental. Research group with a strong international network is proven to be more capable on responding the COVID-19 pandemic which is massive and urgent.

Additionally, we also found that some first authors of the most impactful papers reporting essential oils as therapeutical agents for COVID-19 are new to essential oil research. This indicates that COVID-19 pandemic has attracted researchers to study essential oils for the first time. Though their little experience in essential oil research, but their works could secure a high number of citations. A scientometric study revealed that COVID-19-related papers have higher citation rates as compared with non-COVID-19 papers [43]. Rapid publication and citation of scientific paper covering COVID-19 themes have also been notified another report [44]. In another case, the lead author who have long experience in essential oil research would collaborate with researchers who have different expertise. This suggests the necessity of inter-disciplinary collaboration in performing new research, especially for research involving novel pathogens such as SARS-CoV-2.

### 4.2 Can we use essential oils to manage COVID-19?

There is limited evidence on essential oils efficacies as therapeutical agents for COVID-19. A clinical trial has been conducted to test the efficacy of Ayurvedic medication comprised of various essential oils in managing COVID-19 [45]. Higher recovery and viral clearance rates were observed in the Ayurvedic essential oils arm as compared to standard treatment [45]. Essential oils extracted from *Thymus vulgaris*, *Citrus sinensis*, *Eugenia caryophyllus*, and *Boswellia carterii* were able to assist faster fatigue resolution post-COVID-19, evidenced by a randomized, double-blinded, and placebo-controlled clinical trial [46]. Inhalation of nebulized mixture of several essential oils was found to reduced anosmia and dysgeusia in COVID-19 patients, but no effects were observed in terms of the viral clearance [47]. In another clinical trial, gargling povidone-iodine mixed with essential oils was found efficacious to shorten the period for viral clearance [48]. Some other studies have used this strategy of using essential oil in mouthwash formulation [[49], [50], [51], [52]]. Despite growing evidence supports the efficacy of essential oils as complementary treatment COVID-19, numbers of patients recruited in each clinical trials were too small to reach any clinical implications.

It is still unknown if essential oils could play a role in inhibiting the endocytosis of SARS-CoV-2. Enzyme inhibition assay suggests that several essential oils including eucalyptol, menthol, and rosemary oil could inhibit the activity of angiotensin converting enzyme 2 (ACE2) and stipulated to also inhibit the viral entry [[53], [54], [55]]. Spike protein of SARS-CoV-2 binds to human ACE2, anchored on the surface of cells to gain entry [56]. Lemon and geranium oils were reported efficacious not only in downregulating the expression of ACE2 but also the expression of transmembrane protease serine 2 (TMPRSS2) [35]. An in vitro study employing pseudo-SARS-CoV-2 reported the ability of β-caryophyllene (a volatile commonly compound found in various essential oils) in targeting the interaction between spike protein – ACE2 [57]. However, the same studies also reported worrying findings that several essential oils counterproductively promote the viral entry [57]. Moreover, screening of 30 essential oils proved that only several essential oils are potential to exert antiviral activities [35].

It is worth mentioning that essential oils do not only aim for viral infection or replication, but they also target inflammatory reactions caused by the SARS-CoV-2 infection. Elevated release of interleukin(IL)-6, IL-7, and tumor necrosis factor (TNF) have been recorded as the consequence of innate immune response upon the viral infection [58]. Overproduction of these pro-inflammatory factors could lead to the cytokine storm which contributes to the disease progression into higher severity and even death [59]. In this light, *C. clementine* essential oil has been witnessed to improve TNF-α and IL-6 in SARS-CoV-2-infected huh-7 cells [60]. Limonene, a major constituent of *C. clementine* essential oil, has been reported to reduce the production of pro-inflammatory cytokines [61,62]. Other essential oil that has been reported to reduce the production of pro-inflammatory cytokines is eucalyptus oil (mainly comprised of eucalyptol) [63]. Interestingly, the study also revealed that the essential oil activity of reducing the cytokine levels does not interfere with the phagocytic activities of monocytes and macrophages [63].

### 4.3 Earlier research of antiviral potentials of essential oils

Researchers have explored the antiviral potentials of essential oils since the late 1980s. A research group reported the investigation results of essential oils as plant protectants against potato virus X, tobacco mosaic virus, and tobacco ring spot virus in 1989 [64]. In later dates, reports on protective abilities of various essential oils against plant viruses were published [65]. Antiviral potential of essential oil against herpes simplex virus 1 (HSV-1) has been reported since 1997 by a study using *Salvia fructicosa* essential oil [66]. The constituent of many essential oils, isoborneol, had been shown to exert antiviral activities against HSV-1, based on a report published in 1999 [67]. During the same year, a paper reported that essential oil of *Santalum album* L. could inhibit the replication of HSV-1, yet the remaining non-virucidal [68]. Inhibition of endocytosis of HSV-1 and -2 was achieved by essential oil produced by *Santolina insularis*, reported by a study in 2000 [69].

There are numerous research articles reporting the antiviral activities of essential oils within the span of 2014–2019. The antiviral research of essential oil covers a broad spectrum of viruses started from plant-infecting viruses [70] up to human-infecting viruses [[71], [72], [73], [74], [75], [76]]. Essential oil activities against HSV-1 and influenza viruses were among the most investigated [[71], [72], [73],^75^,^76^]. Taken altogether, antiviral activities of essential oils have been long explored. However, the research did not receive adequate spotlight in 2014–2019 period. Moreover, activities of essential oils against coronaviruses were previously underreported. In this present study, antiviral research has been suggested to appear prominent only after the COVID-19 pandemic started. Therefore, the COVID-19 pandemic is the moment for researchers to exhaust the antiviral studies of essential oils.

### 4.4 Current challenges and new trajectories

Analysis on the keywords from the published literatures revealed that main focus of essential oil research was to investigate the *anti*-SARS-CoV-2 potential, where most of the studies performed the screening based on ACE2 inhibition [35,[53], [54], [55],^77^]. Computational simulations, including the most popular one – molecular docking, have become the main modality to screen the antiviral potential of essential oils against SARS-CoV-2 [[78], [79], [80], [81], [82], [83]]. Computational approaches might be useful, especially to overcome the shortcoming of well-established research protocol to screen the antiviral candidates during the wake of the novel virus outbreak. However, computational simulation is solely dependent on ligand–protein interaction while neglecting other antiviral pathways and various physiological responses. Disagreement between in silico data and in vitro data has been witnessed in a study screening phytocompounds for inhibiting human ACE2-spike protein interaction [84]. To provide a more accurate data, researchers have used several cancer cell lines used to study the *anti*-SARS-CoV-2 potential of essential oils, such as Vero-E6 cells [60,85], Huh7 cells [60], HEK293 cells [57], and HT-29 cells [35]. Still, the results are limited to infer the real potential of essential oils in normal cells due to the significant physiological difference between normal and cancer cells. Moreover, in-vivo and clinical studies are mandatory before drawing any conclusions regarding the benefits of essential oils for infectious diseases.

From the laboratory findings, hence their non-definitive nature, several essential oils have been proven to yield *anti*-SARS-CoV-2 and anti-inflammatory activities. However, current research suggests that only some of the essential oils are active as *anti*-SARS-CoV-2. As reported previously, out of 30 essential oils, only two were found potential as ACE2 inhibitors [35]. Another report even suggested the possibility of several essential oils to act as the viral entry promotors [57]. As in the clinical trials, essential oils were often used in a mixed formulation. Moreover, the fate and bioavailability essential oils have not been well-documented. The efficacy of essential oils to facilitate early viral clearance and symptom resolution should be subjected to their metabolites.

Based on the explanations above, we recommend these followings for new research trajectories:

a. Computational or in silico techniques should be enhanced to overcome its current limitations. Implementation of machine learning and deep learning could assist the development of these techniques.
b. In vitro model, including the cell culture and viral designs, should be further developed to mimic the physiological response in human body. Alternatively, 3D model of lung tissue with close similarity to that in humans is available. Yet, its use might be limited due to its expensive price, implying the necessity for further development to lower the price.
c. Clinical trial studies have to be carried out on the single component of essential oil in managing COVID-19.
d. Studies on antiviral activities of essential oils along with their immunomodulatory activities should be performed on the metabolites derived from rapid metabolism of essential oils in the body.

### 4.5 Strengths and limitations

Evidence provided from the bibliometric analysis relies on the published research, while there are findings about ‘essential oils for COVID-19’ that have not been published. Additionally, the present study only used a single database (Scopus), and not all publications are indexed in the database. Validation using more straightforward experimental methods, such as interview or direct observation, should be performed in the future. Regardless, bibliometric is a strong tool that can quickly present information on the research landscape. Moreover, we are the first to utilize bibliometric approach to observe the impact of COVID-19 on certain research theme (in this case essential oil).

## 5 Conclusions

COVID-19 pandemic does not significantly shift the essential oil research progress, but it is more likely to hint the antiviral potential of essential oils. During the COVID-19 pandemic the research progresses to cover several crucial topics including ‘anticancer’ and ‘poultry supplement’. Research on essential oils experience a tremendous growth in many countries from different continents. Research groups with a strong international and inter-disciplinary collaboration have more capability to response to abrupt situations such as this current pandemic. More importantly, COVID-19 pandemic has shed a new spotlight on the research of essential oils regarding their antiviral potential. Indeed, there is growing evidence on essential oils efficacy as *anti*-SARS-CoV-2 agent. Research design development is required to give meaningful data about essential oil potentials as COVID-19 therapeutical agents. We particularly recommend the investigation of single component essential oil in clinical trial settings.

## Author contribution statement

All authors listed have significantly contributed to the development and the writing of this article.

## Data availability statement

Data will be made available on request.

## Author contribution statement

All authors listed have significantly contributed to the development and the writing of this article.

## Funding

This study received no external fund.

## Declaration of competing interest

The authors declare that they have no known competing financial interests or personal relationships that could have appeared to influence the work reported in this paper.
