# Supplementary material for: COVID-19 pandemic sheds a new research spotlight on antiviral potential of essential oils – A bibliometric study
Source: Heliyon. 2023 Jun 29;9(7):e17703. doi: 10.1016/j.heliyon.2023.e17703 (PMC10338973; doi:10.1016/j.heliyon.2023.e17703)
Supplement: Multimedia component 1 [file mmc1.docx]

## SUPPLEMENTARY FILES

Initial search via Scopus database "Aromatherapy" OR "Essential Oil" OR "Aromatic Plant”:

77539 papers retrieved

Excluded due year published (n=53232)

Papers screened by document type:

24307 papers retrieved

Excluded due to document type, publication stage (n=84)

Papers screened by document type, publication stage:

24223 papers retrieved

Subject areas exclusion
(n=4528)

Papers screened by subject areas limitation:

19695 papers retrieved

Final papers included:
n=18670

Excluded due to language (n=1025)

Figure S1. Flowchart of essential oils literature search pre-COVID-19

Initial search via Scopus database "Aromatherapy" OR "Essential Oil" OR "Aromatic Plant”:

77539 papers retrieved

Excluded due year published (n=58238)

Papers screened by year published:

19211 papers retrieved

Excluded due to document type and publication stage (n=75)

Papers screened by document type, publication stage:

19136 papers retrieved

Subject areas exclusion
(n=1750)

Papers screened by subject areas limitation:

17386 papers retrieved

Excluded due to language (n=794)

Final papers included:
n=16592

Figure S2. Flowchart of essential oils literature search during COVID-19

Initial search via Scopus database ("Aromatherapy" OR "Essential Oil" OR "Aromatic Plant”) AND (“COVID” OR “Sars-cov-2” OR “Coronavirus”):

333 papers retrieved

Papers screened by document type:

325 papers retrieved

Excluded due year published (n=8)

Excluded due to document type and publication stage (n=2)

Papers screened by document type, publication stage:

323 papers retrieved

Subject areas exclusion
(n=36)

Papers screened by subject areas limitation:

287 papers retrieved

Final papers included:
n=281

Excluded due to language (n=6)

Figure S3. Flowchart of essential oils & COVID-19 literature search

Table S1. Top 10 most cited article of aromatherapy pre-COVID-19

| **Rank** | **Title** | **Author(s)** | **Year of Publication** | **Times of Citations** | **DOI** |
| --- | --- | --- | --- | --- | --- |
| 1 | Essential oils as antimicrobials in food systems - A review | Calo et al. | 2015 | 699 | [10.1016/j.foodcont.2014.12.040](https://doi.org/10.1016/j.foodcont.2014.12.040) |
| 2 | A status review on the medicinal properties of essential oils | Raut, J. & Karuppayil, S. | 2014 | 692 | 10.1016/j.indcrop.2014.05.055 |
| 3 | Essential Oils as Ecofriendly Biopesticides? Challenges and Constraints | Pavela, R. & Benelli, G. | 2016 | 549 | [10.1016/j.tplants.2016.10.005](https://doi.org/10.1016/j.tplants.2016.10.005) |
| 4 | Essential oils: From extraction to encapsulation | Ashabani et al. | 2015 | 523 | [10.1016/j.ijpharm.2014.12.069](https://doi.org/10.1016/j.ijpharm.2014.12.069) |
| 5 | Essential oils as additives in biodegradable films and coatings for active food packaging | Atarés, L. & Chiralt, A. | 2016 | 510 | [10.1016/j.tifs.2015.12.001](https://doi.org/10.1016/j.tifs.2015.12.001) |
| 6 | Antibacterial activity and mechanism of cinnamon essential oil against Escherichia coli and Staphylococcus aureus | Zhang et al. | 2016 | 456 | [10.1016/j.foodcont.2015.05.032](https://doi.org/10.1016/j.foodcont.2015.05.032) |
| 7 | Essential oils for the development of eco-friendly mosquito larvicides: A review | Pavela, R. | 2015 | 454 | [10.1016/j.indcrop.2015.06.050](https://doi.org/10.1016/j.indcrop.2015.06.050) |
| 8 | Antimicrobial properties of plant essential oils against human pathogens and their mode of action: An updated review | Swamy et al. | 2016 | 453 | [10.1155/2016/3012462](https://doi.org/10.1155/2016/3012462) |
| 9 | Recent advances on antimicrobial wound dressing: A review | Simões et al. | 2018 | 452 | [10.1016/j.ejpb.2018.02.022](https://doi.org/10.1016/j.ejpb.2018.02.022) |
| 10 | Microencapsulation of Oils: A Comprehensive Review of Benefits, Techniques, and Applications | Bakry et al. | 2016 | 449 | [10.1111/1541-4337.12179](https://doi.org/10.1111/1541-4337.12179) |

Table S2. Top 10 most cited article of aromatherapy during-COVID-19

| **Rank** | **Title** | **Author(s)** | **Year of Publication** | **Times of Citations** | **DOI** |
| --- | --- | --- | --- | --- | --- |
| 1 | Antibacterial biohybrid nanofibers for wound dressings | Homaeigohar, S. & Boccaccini, A.R. | 2020 | 235 | [10.1016/j.actbio.2020.02.022](https://doi.org/10.1016/j.actbio.2020.02.022) |
| 2 | Chemical constituents and pharmacological activities of garlic (*Allium sativum* L.): A review | Batiha et al. | 2020 | 212 | [10.3390/nu12030872](https://doi.org/10.3390/nu12030872) |
| 3 | Chitosan nanoparticles loaded with clove essential oil: Characterization, antioxidant and antibacterial activities | Hadidi et al. | 2020 | 198 | [10.1016/j.carbpol.2020.116075](https://doi.org/10.1016/j.carbpol.2020.116075) |
| 4 | Botanical insecticides in the twenty-first century-fulfilling their promise? | Isman, M.B. | 2020 | 188 | [10.1146/annurev-ento-011019-025010](https://doi.org/10.1146/annurev-ento-011019-025010) |
| 5 | *Syzygium aromaticum* l. (myrtaceae): Traditional uses, bioactive chemical constituents, pharmacological and toxicological activities | Batiha et al. | 2020 | 171 | [10.3390/biom10020202](https://doi.org/10.3390/biom10020202) |
| 6 | Edible films/coating with tailored properties for active packaging of meat, fish and derived products | Umaraw et al. | 2020 | 163 | [10.1016/j.tifs.2020.01.032](https://doi.org/10.1016/j.tifs.2020.01.032) |
| 7 | Essential oils: A promising eco-friendly food preservative | Falleh et al. | 2020 | 154 | [10.1016/j.foodchem.2020.127268](https://doi.org/10.1016/j.foodchem.2020.127268) |
| 8 | An overview of micro-and nanoemulsions as vehicles for essential oils: Formulation, preparation and stability | Pavoni et al. | 2020 | 153 | [10.3390/nano10010135](https://doi.org/10.3390/nano10010135) |
| 9 | Investigation into SARS-CoV-2 Resistance of Compounds in Garlic Essential Oil | Thuy et al. | 2020 | 152 | [10.1021/acsomega.0c00772](https://doi.org/10.1021/acsomega.0c00772) |
| 10 | Titanium dioxide nanoparticles (TiO_2_ NPs) promote growth and ameliorate salinity stress effects on essential oil profile and biochemical attributes of *Dracocephalum moldavica* | Gohari et al. | 2020 | 147 | [10.1038/s41598-020-57794-1](https://doi.org/10.1038/s41598-020-57794-1) |
